# Hyperbaric Oxygen Therapy in the Treatment of Crohn’s Disease

**DOI:** 10.3390/healthcare13020128

**Published:** 2025-01-11

**Authors:** Jure Krstulović, Goran Augustin, Ivan Romić, Ante Tavra, Franko Batinović, Zrinka Hrgović

**Affiliations:** 1 School of Medicine, University of Split, Šoltanska 2, 21000 Split, Croatia; jure.krstulovic@mefst.hr (J.K.); ante.tavra@mefst.hr (A.T.); franko.batinovic@mefst.hr (F.B.); zrinka.hrgovic@mefst.hr (Z.H.); 2 Department of Surgery, University Hospital of Split, Spinčićeva 1, 21000 Split, Croatia; 3 Department of Surgery, University Hospital Centre Zagreb, Kišpatićeva 12, 10000 Zagreb, Croatia; ivan.romic@kbc-zagreb.hr; 4 Department of Otorhinolaryngology, University Hospital of Split, Spinčićeva 1, 21000 Split, Croatia

**Keywords:** hyperbaric oxygen therapy, inflammation, inflammatory bowel disease, Crohn’s disease, Croatia

## Abstract

**Background/Objectives**: Our study describes hyperbaric oxygen therapy (HBOT) as an additional therapy in the conservative treatment of Crohn’s disease (CD) and its benefit in the early postoperative period to prevent surgical complications and improve gastrointestinal motility. **Methods**: This retrospective study evaluated HBOT in patients hospitalized at the Clinical Hospital Center Split for complications of CD between 2015 and 2020. Patients (N = 61) aged 19 to 67 with perianal fistulas, abscesses, fistulas, obstruction, stenosis, or bleeding were included, excluding those with ulcerative colitis or requiring intensive care. Patients were retrospectively divided into conservatively and surgically treated groups, and HBOT was administered over 15–25 days, with treatment lasting 60 min at 2.2 absolute atmospheres (ATA). We analyzed treatment outcomes between the HBOT-treated surgical and conservative groups and compared patients treated with HBOT to a cohort from the preceding five years who did not receive HBOT. **Results**: We treated 61 CD patients with HBOT, including 34 conservatively and 27 surgically treated patients. HBOT significantly reduced disease activity indices (311.7 ± 59.1 vs. 114 ± 29.8; 203.6 ± 24.1 vs. 83.8 ± 15, for conservatively treated patients, and 352.8 ± 45.7 vs. 109 ± 22.8; 270.4 ± 19.7 vs. 140.3 ± 10.6 for surgically treated patients) and accelerated bowel peristalsis recovery, with 94.1% of conservatively treated patients achieving remission. Comparison with a historical cohort showed faster recovery and improved outcomes in the HBOT group. **Conclusions**: HBOT is useful in postponing or avoiding surgical treatment, and in operated patients, it improves postoperative recovery and reduces the rate of postoperative complications.

## 1. Introduction

Inflammatory Bowel Diseases (IBDs) are chronic, relapsing diseases characterized by recurrent inflammation of the gastrointestinal tract, bloody diarrhea, abdominal pain, and nonspecific symptoms such as fever and weight loss [1,2,3,4]. One subtype of IBD is Crohn’s disease (CD), a chronic, transmural, nonspecific, granulomatous inflammatory disease of unclear etiology and pathogenesis, which makes the course and prognosis difficult to predict [5]. Although the disease typically affects the terminal ileum and the right colon, inflammation can involve the entire gastrointestinal tract [6].

The etiology of this disease is established as multifactorial. Still, some research suggests that it is caused by an imbalance between pro-inflammatory and anti-inflammatory factors due to the interaction between genetics, a weakened immune response, and environmental factors [2,3,4,5]. Genetic predisposition has a significant role in the etiology of CD. Still, the distribution of the disease within families is complex and cannot be classified by simple Mendelian inheritance laws [7]. Therefore, it results from a complex interaction between genetic predisposition, such as the X chromosome in women, which establishes a fundamental susceptibility, and environmental factors, including smoking and early life events such as cesarean section, a high level of hygiene, and repeated antibiotic exposure, that may trigger the disease’s genesis or intensify its progression [4,8,9]. Also, there is the emerging role of the melanocortin system, particularly the melanocortin-3 receptor (MC3R) and melanocortin-5 receptor (MC5R), critical regulators of immune activity through their interaction with α-melanocyte-stimulating hormone (α-MSH) and adrenocorticotropic hormone (ACTH), whose degree of expression in the colons of IBD patients points to a possible correlation with the degree of disease activity [10]. Furthermore, the recent review by Gravina emphasizes the importance of the melanocortin system in IBD, suggesting that the dysregulation of this system may contribute to the inflammatory processes observed in conditions like CD [11]. This suggests that these receptors could be biomarkers for disease progression and therapeutic targets [10,11].

Recent studies also emphasize the role of intestinal barrier dysregulation and changes in gut flora in the pathophysiology of CD [12]. Normally, the intestinal epithelium is in a state of “physiological hypoxia”, which may worsen hypoxic conditions with inflammation [13], leading to oxidative stress (OS) [14]. Activated macrophages play a key role in the disease process, producing pro-inflammatory cytokines, including TNF-α and interleukins (IL-6 and IL-8) [15]. OS, driven by an excess of reactive oxygen species (ROS), occurs locally and systematically in individuals with CD and seems to be associated with dysbiosis and immune response imbalance [16,17]. Furthermore, it is one of the main causes of tissue damage and fibrosis that define CD and significantly raises IL-8, chemokines, and neutrophile production [18]. In patients with CD, immune mononuclear cells in inflamed mucosa and submucosa produce many potentially harmful ROS, such as H_2_O_2_. At the same time, catalase activity is consistently suppressed, meaning that cells have impaired antioxidant defense [19,20]. Consequently, OS provides a route to bacterial invasion through the damaged mucosal layer, further increasing the immunological response and exacerbating disease progression [21]. The pathophysiology of CD is significantly influenced by epigenetic mechanisms, particularly microRNAs [22]. Furthermore, OS-related genes that contribute to the development of CD are controlled by DNA methylation, gene expression, and host interaction with gut microbiota [23]. The levels of intestinal nitric oxide (NO) are also elevated in some IBD patients, which may lead to increased tissue damage [24].

Given that IBD is not clinically curable, the available therapies aim to improve the quality of life, alleviate symptoms, reduce the incidence of complications, and slow disease progression [5,6,25]. The treatment of CD is typically pharmacological, involving steroids, immunomodulators, and biological agents. The goal is to achieve clinical remission and mucosal healing [5]. Nevertheless, half of CD patients still require surgery during their lifetime [2,3,4,5,26,27]. The surgical treatment of CD is indicated for complications such as obstruction, free perforation, abscesses, symptomatic fistulas, bleeding, and cancers [2,3,4,5,26,27,28]. Due to the negative impact of immunomodulators on immune function, the adverse events associated with long-term corticosteroid use, and the fact that more than 40% of patients do not respond to TNF inhibitors or develop late resistance to the drugs, newer non-pharmacological approaches to treating IBD have emerged, one of which is hyperbaric oxygen therapy (HBOT) [29,30,31].

HBOT involves inhaling 100% oxygen for 60–90 min at a higher than normal atmospheric pressure (often 2.0–2.5 absolute atmosphere (ATA)), which increases plasma and tissue oxygen levels and reduces hypoxia [32]. As we have said, hypoxia promotes the development and maintenance of inflammation in CD. A few studies have highlighted HBOT as a method to improve tissue health and blood oxygen levels [33,34]. HBOT enhances tissue oxygenation, alleviates hypoxia, and modifies inflammatory pathways in CD patients [35]. Furthermore, other mechanisms underlying the therapeutic effect were found in experiments with mice, showing that HBOT enhanced the activity of the antioxidant enzymes glutathione peroxidase (GPx) and superoxide dismutase (SOD), which reduced oxidative stress. Additionally, it enhanced the anti-inflammatory cytokines IL-4 and IL-10 while decreasing the pro-inflammatory cytokines IFN-γ, IL-12, IL-17, and TNF-α [36]. These described signaling pathways and inflammatory cytokines play a significant role in the intestinal microenvironment in the process of inflammation activation in IBD [1,3]. HBOT was first introduced as an adjunctive treatment for CD in 1989 when Brady and colleagues reported its effectiveness in healing severe perineal CD, particularly in resolving complications and promoting the healing of intestinal mucosal lesions [33]. Other complications of CD, such as perianal disease, enterocutaneous fistulas, and pyoderma gangrenosum, were also successfully treated with HBOT [37,38,39]. Also, combined with vedolizumab, it permanently disrupts inflammatory stimuli, promoting tissue regeneration and healing small bowel stenosis with an interintestinal fistula [40]. Furthermore, perianal fistulae, including rectovaginal fistulae, heal quickly when infliximab is used with anti-MAP treatment and HBOT [41].

According to the Undersea and Hyperbaric Medical Society (UHMS), clinical practice involves a patient lying in a sealed chamber and breathing oxygen at atmospheric pressures between 2 and 3 ATA, resulting in elevated blood (hyperoxemia) and tissue (hyperoxia) oxygen levels [42,43]. Depending on the indication, treatments typically last one to two hours and can be administered one to three times a day [44]. When normobaric air is inhaled, the arterial pO_2_ in the tissues is roughly 100 and 55 mmHg. However, arterial pO_2_ can rise to approximately 2000 mmHg and 500 mmHg in the tissues when inhaling 100% oxygen at 3 ATA [45].

The UHMS currently accepts 15 approved indications: air or gas embolism, central retinal artery occlusion, carbon monoxide poisoning, clostridial myonecrosis (gas gangrene), compromised surgical grafts and flaps, crush injuries/skeletal muscle compartment syndrome/acute arterial insufficiency, decompression sickness, intracranial abscess, necrotizing soft tissue infections, exceptional blood loss anemia, specific acute thermal burns, and idiopathic sudden sensorineural hearing loss (urgent) [46]. While it is not an established indication, research on HBOT as a supplement to traditional treatment for IBD has increased recently. HBOT may reduce oxidative stress, stimulate colonic stem cell differentiation and attract repair-related cells, limit mucosal inflammation, promote ulcer healing, influence gut bacteria, and lower the incidence of IBD complications [47,48].

This study aims to provide a single-center experience with HBOT as an adjunct method for CD complications and to review the literature on the development of HBOT in general and its use in IBD, focusing on its role in the early postoperative period.

## 2. Materials and Methods

**Figure 1:**
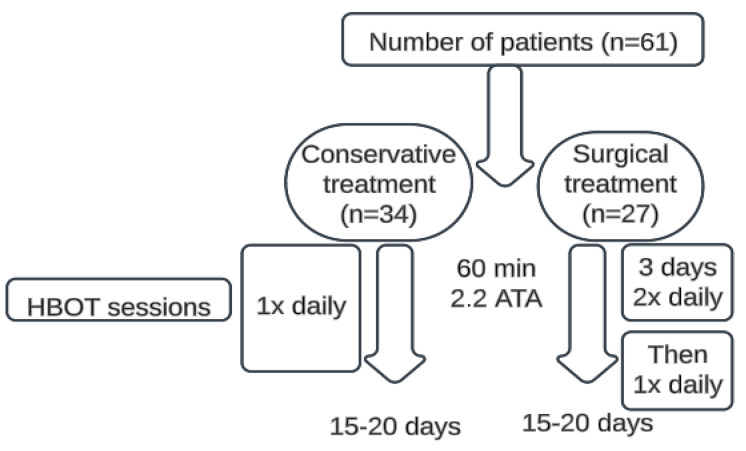
HBOT protocol in the treatment of Crohn’s disease.

This is a retrospective study of HBOT used to treat patients hospitalized at the University Hospital of Split due to CD complications. The University Hospital of Split has access to a hyperbaric chamber through collaboration with the Maritime Institute, allowing HBOT use. The HBOT was conducted between 2015 and 2020 as a previously validated treatment for CD. Informed consent was waived for this retrospective study as all data were anonymized and collected as part of routine clinical practice, in compliance with ethical guidelines and the institutional Ethics Committee approval of the University Hospital of Split (2181-147/01/06/M.S.-22-02). The complications of active moderate to severe CD were defined as a perianal fistulizing disease with abscesses, enterocutaneous fistulas, intra-abdominal abscesses, terminal ileitis, obstruction, stenosis, or perforation of the small/large intestine, and intestinal bleeding. Both surgically and conservatively treated patients were included, with surgical patients further classified by Greenstein’s classification into perforating and non-perforating types. Hyperbaric oxygenation was administered for 15–20 days, with treatments lasting 60 min at 2.2 ATA. Conservatively treated patients received one treatment per day, while surgically treated patients received two treatments per day for the first three postoperative days, followed by one treatment per day. Patients received 15–25 treatments (Figure 1).

Only adult patients (N = 61) aged 19 to 67 were included if they were in an active state with a baseline “Crohn’s Disease Activity Index” (CDAI) score of 220–450, which represents moderate to severe disease. Patients with ulcerative colitis or who required respiratory support or intensive care during the first three postoperative days were excluded. Patients were excluded from this study if they had any medical condition contraindicating HBOT. Follow-up was at least 12 months.

Data were retrospectively collected from the documentation maintained during the hyperbaric oxygenation procedure. They included demographic data and clinical characteristics: previous operations, disease type for surgical patients, disease activity measured by the CDAI and the van Hees index, CRP levels, and ASA score.

The primary outcome was to present changes in disease activity indices (CDAI and van Hees index) and show if there is clinical remission, defined as CDAI cut-off <150. Secondary outcomes included the length of hospitalization, the surgery rate in initially conservatively treated patients, morbidity, mortality, and the time to return bowel peristalsis. Additionally, in conservatively treated patients, treatment success was evaluated through laboratory findings and the radiological regression of characteristic signs of CD, including mucosal healing, mesenteric infiltration reduction, bowel passage restoration, and the absence of interintestinal fistulas. The secondary outcome was also to assess the effectiveness of HBOT by comparing the results with patients from the previous five-year period at the University Hospital of Split (n = 76) treated for the same indications.

Categorical variables were presented as ratios and percentages and compared using the Chi-square test. Continuous variables were presented as mean ± standard deviation (for parametric data) or median and interquartile range (for non-parametric data) and compared using the *t*-test or Mann–Whitney U test. A *p*-value < 0.05 was considered statistically significant. All data were analyzed using SPSS v27^®^ (IBM, Armonk, NY, USA).

## 3. Results

We treated 61 patients with CD using HBOT. Of these, 28 were men (45.9%) and 33 were women (54.1%). As mentioned earlier, this distribution reflects the typical demographics of CD, which is more often diagnosed in women. The average age was 32.7 years (range 19–67). Conservative treatment was administered to 34 patients (55.7%), while 27 patients (44.3%) underwent surgery (Table 1). Before admission, all patients had confirmed CD and were treated by a gastroenterologist who employed conservative measures (aminosalicylates, metronidazole, biologic therapy, and dietary interventions). Nine patients (14%) had undergone prior surgery for CD. There was no significant difference in anthropometric characteristics, ASA score, or the number of previous surgeries (*p* > 0.05). Comparative analysis for continuous non-parametric variables was performed with the Mann–Whitney U test and parametric variables with Student’s *t*-test. The comparison of categorical variables was performed with the Chi-square test. In contrast, the two-way Fisher’s exact test was used in cases where the frequency of the observed parameter was low.

During HBOT, the only specific therapy for CD was 500 mg of metronidazole three times daily. Among surgically treated patients, 12 (44.4%) had the perforating (P) type of disease, while only 6 (17.6%) of the conservatively treated patients had the P-type, which was a statistically significant difference (*p* < 0.005) (Table 1).

**Figure 2:**
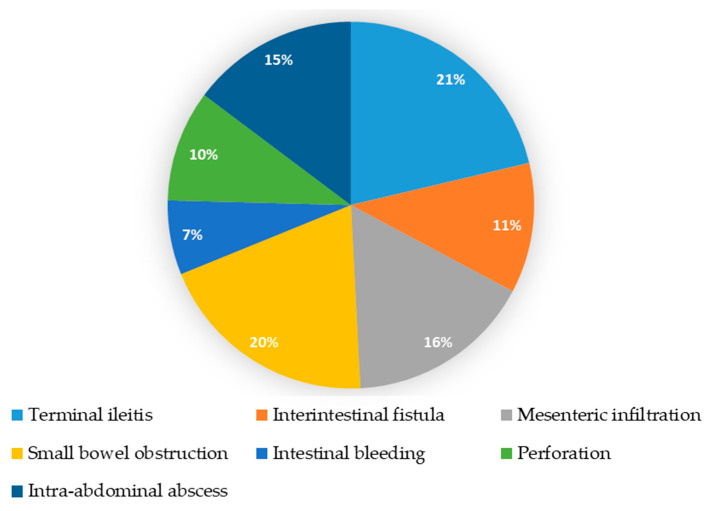
Crohn’s disease complications as a major cause of patient hospitalization. Each pie stake is associated with a percentage of 61 patients hospitalized for a certain complication.

Figure 2 shows Crohn’s disease complications as a major cause of patient hospitalization, which differed between the groups (Table 1). Among conservatively treated patients, the most common criteria were terminal ileitis (35%) and mesenteric infiltration (29%). In contrast, in the surgical group, the main criteria for surgery were small bowel obstruction (29.6%) and the perforation of a hollow organ (22.2%).

**Figure 3:**
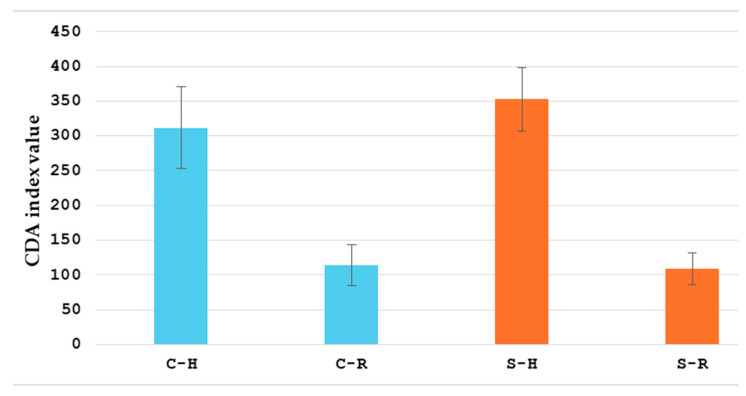
CDA index values for patients treated conservatively (blue) and surgically (orange). C-H: CDA index value on the first day of hospital admission for patients that were treated conservatively; C-R: CDA index 30 days post-treatment for patients that were treated conservatively; S-H: CDA index on the first day of hospital admission for patients that were treated surgically; S-R: CDA index 30 days post-treatment for patients that were treated surgically.

**Figure 4:**
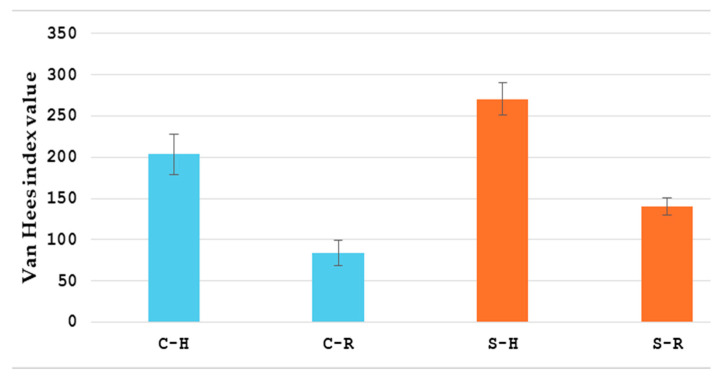
Van Hees index values for patients treated conservatively (blue) and surgically (orange). C-H: Van Hees index value on the first day of hospital admission for patients that were treated conservatively; C-R: Van Hees index 30 days post-treatment for patients that were treated conservatively; S-H: Van Hees index on the first day of hospital admission for patients that were treated surgically; S-R: Van Hees index 30 days post-treatment for patients that were treated surgically.

Table 2 shows the clinical outcomes. Conservatively treated patients achieved clinical remission and the restoration of the bowel passage in 94.1% (32 patients), while 2 patients required surgery due to complications (bowel obstruction and intra-abdominal abscess). Out of these two, one patient had four sessions of HBOT, and another one had twelve sessions of HBOT. In patients with mesenteric infiltration, there was a significant reduction in the infiltration, verified postoperatively by enteroclysis, CT, or ultrasound. Interintestinal fistulas were not detected by enteroclysis after HBOT treatment. The CDAI and van Hees index were measured on the first day of hospital admission and 30 days after the treatment. A significant decrease (*p* < 0.01) in both CDA (311.7 ± 59.1 vs. 114 ± 29.8 for conservatively, and 352.8 ± 45.7 vs. 109 ± 22.8 for surgically treated patients) and van Hees (203.6 ± 24.1 vs. 83.8 ± 15, for conservatively, and 270.4 ± 19.7 vs. 140.3 ± 10.6 for surgically treated patients) indices for both groups was observed (Figure 3 and Figure 4). The CDAI and van Hees index are the leading indicators that show how HBOT is useful in postponing or avoiding surgical treatment in conservatively treated patients. In operated patients, it improves postoperative recovery and reduces the rate of postoperative complications.

Surgically treated patients were managed according to “minimal surgery” principles and standardized indications. Peristalsis resumed in most operated patients after the first two HBOT treatments (85.1%). Peristalsis was verified by stethoscope auscultation, and during morning visits/rounds, patients were also asked to report if they felt bowel movement.

**Figure 5:**
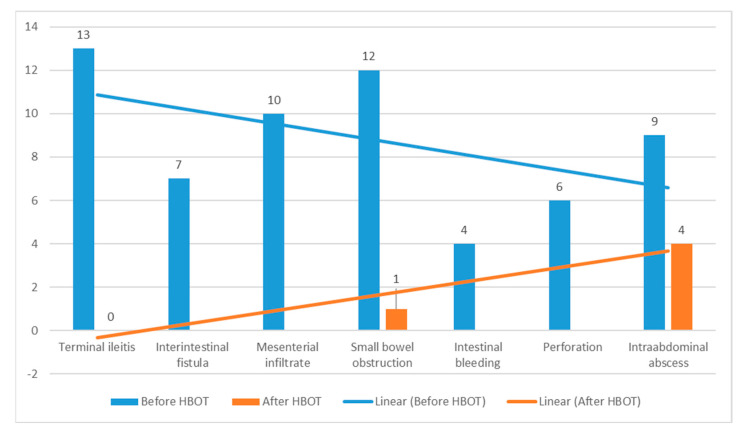
Crohn’s disease complication frequency before and after HBOT.

Early postoperative complications included one surgical wound infection and one case of bronchopneumonia, both managed conservatively, while two patients required surgical revision due to intra-abdominal abscesses. There were no other CD-related complications, such as postoperative fistulas, bleeding, or intestinal obstruction. Figure 5 presents the effect of HBOT on the incidence of CD complications. There was no mortality during the postoperative course or 12 months of follow-up. During the observation period, 16 patients (26.2%) were treated with HBOT oxygen therapy again due to disease relapse, and 4 patients (6.5%) underwent treatment two or more times.

**Figure 6:**
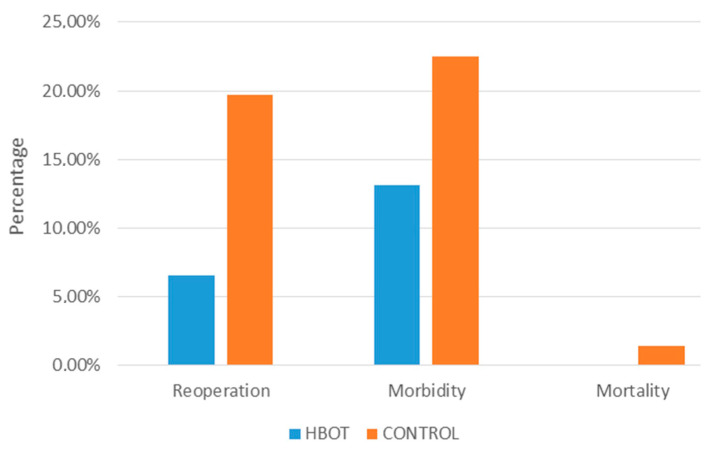
Primary outcomes between the HBOT-treated and untreated groups.

Further analysis compared the demographic and clinical parameters and treatment outcomes of the group treated with HBOT and patients from the five-year period (2020–2015) who were not treated with HBOT (76 patients). the results are shown in Table 3 and Figure 6. The average age and proportion of male patients are similar between the two groups, with *p*-values (0.81 and 0.72, respectively) indicating no significant difference. Without HBOT, no significant difference (*p* < 0.05) was found between 29 surgically and 47 conservatively treated patients. The mean BMIs are also not significantly different (*p* = 0.54). The distribution between perforating and non-perforating types of the disease shows no significant difference (*p* = 0.36). The ASA scores, which assess the physical status of patients, do not show a significant difference between groups (*p*-values 0.82 and 0.73). A significant difference is noted in the mean CRP levels, with the HBOT group having lower levels (*p* < 0.05), suggesting potentially less inflammation or the better management of inflammatory responses with HBOT. Mortality was zero in the HBOT group compared to the non-HBOT group. A significant reduction in the need for reoperation is seen in the HBOT group (*p* = 0.04), suggesting better initial outcomes with HBOT. Patients in the HBOT group had a significantly shorter median hospitalization duration compared to the non-HBOT group (*p* < 0.05), which can imply more efficient treatment or faster recovery with HBOT. There is a lower percentage of morbidity in the HBOT group, but this was not statistically significant (*p* = 0.22). The time to recovery of bowel peristalsis was significantly quicker in the HBOT group (*p* < 0.05), indicating a faster recovery of gut function with HBOT. Both the CDAI (Crohn’s Disease Activity Index) and the van Hees index show greater improvement in the HBOT group with statistically significant differences (*p* < 0.05). So, the groups did not significantly differ in clinical characteristics. However, the patients treated with HBOT experienced a significantly faster recovery of bowel peristalsis, shorter hospitalization duration, and a more significant decrease in disease activity indices than those not treated with HBOT.

## 4. Discussion

In this study, we presented our experience with HBOT as an adjunctive therapeutic method in treating patients hospitalized for acute complications of CD. Our institution used HBOT for both conservatively and surgically treated patients, relying on the results of worldwide studies that showed benefits in treating other inflammatory conditions and complications of IBD.

All patients were compliant, and all organizational measures were successfully implemented to provide HBOT to all consecutive patients during the study period. There were no complications related to the HBOT procedure, and none of the patients withdrew before or during the HBOT protocol.

Surgical patients, as expected, had more severe disease according to standardized indices (CDAI and van Hees index) compared to conservatively treated patients. However, the control group from the previous five-year period did not differ significantly in age, gender, type, or severity of CD complications. This allowed us to perform a reliable comparative analysis. The most significant effect of HBOT was reduced hospital stay and the need for surgical intervention or reoperation in initially surgically treated patients. This is extremely important because the treatment of CD complications aims to avoid bowel resections due to the potential risk of short bowel syndrome. Therefore, conservative and medical therapy represents the foundation of treating CD and its complications while preventing or delaying surgery when possible.

Our results demonstrate the benefits of HBOT in conservatively and surgically treated patients. The reduction in hospital stay, lower complication rates, and positive impact on disease activity suggest that HBOT should become a standard adjunctive therapy for patients with acute complications of CD.

It is worth mentioning the potential role of HBOT in enhancing physical performance and recovery, particularly in patients with CD. The “BE-FIT-IBD” study revealed that IBD patients often experience significantly low levels of physical activity with several barriers that contribute to that inactivity, such as fatigue, disease activity, and psychological factors [49,50]. The postoperative morbidity rate was lower in HBOT-treated patients (13.1% vs. 21.0%, *p* = 0.22), which is an important, though not statistically significant, difference. Mortality could not be analyzed due to the low mortality rate, with only one death in the earlier group due to multiple comorbidities and septic complications.

Additionally, our study included individual cases with clinical and radiological signs of subocclusion who, after repeated HBOT treatments, achieved normal bowel passage and resumed oral feeding. In one patient with an interintestinal fistula, clinical improvement was observed 13 days after treatment, and the absence of the fistula was verified by enteroclysis. These examples suggest that some previous surgical indications may be managed conservatively using HBOT. The effect of HBOT on bowel passage is of particular interest to surgeons, as there is a hypothesis that HBOT may contribute to the non-operative treatment of adhesive bowel obstruction, which is ideal, especially for patients who have recently undergone surgery for the same condition. Fontaine first described the direct effect of high atmospheric pressure on a closed intestinal loop containing gas. He discovered that hyperbaric pressure reduces bowel distension following Boyle’s law, suggesting that this therapy may improve postoperative paralytic ileus and adhesive bowel obstruction [51]. In our study, peristalsis resumed in over 80% of operated patients within the first two postoperative days.

Therefore, it is justified and beneficial to use HBOT as an adjunctive therapy for conservatively treated patients and as a supportive therapy in the early postoperative period. However, further prospective clinical studies on more patients are needed, considering other parameters such as bowel absorption, laboratory parameters, and histochemical and immunohistochemical findings. The availability of HBOT chambers presents a challenge, and improving access to hyperbaric chambers in select hospitals could enhance treatment options for patients with IBD and certain vascular, neurological, and dermatological conditions.

Since 1813, when Combe and Saunders first described changes in the ileum associated with CD, the clinical manifestations of this disease have been thoroughly researched. Still, its multifactorial etiology and pathogenesis remain undefined [52]. Crohn, Ginzburg, and Oppenheimer published a historical paper in 1933, and since then, treatment principles have changed several times [53]. The most significant changes have occurred in the surgical approach and in developing more effective anti-inflammatory drugs [2,6,7]. The current approach is based on conservative therapy, with surgery reserved only for complications. Additionally, surgical treatment should follow the principles of so-called “minimal surgery” to avoid excessive bowel resections, delaying surgery for the later, advanced stages of the disease [5,7,54].

Funnayama et al. used histometric measurements of arteries and arterioles to show that atrophic changes in a particular layer of blood vessels (tunica media) lead to subsequent thrombosis, ischemia, and fibrosis. This finding justifies the use of HBOT in treating CD [55]. The method is not a cure, as the cause of changes in the terminal branches of intestinal blood vessels is still unknown, making it impossible to save irreversibly damaged intestines. Future clinical and basic research on CD will aim to improve treatment, prevent exacerbations, and delay small intestine resections. The therapy that could achieve these goals is HBOT, which has been successfully used in many other diseases and conditions where its safety and efficacy have already been proven [25,31].

There are some limitations to our study. First, it is a retrospective study, which made patient randomization impossible, but selection bias was minimized by clearly defining inclusion criteria. However, the groups were not uniform according to disease severity, and there was a discrepancy in HBOT sessions during the first three days between the groups. Second, a small sample size could not reach statistically significant differences in some analyses. Third, the control group in the comparative section of the study comprised patients from an earlier period. Other advancements in treatment possibly contributed to better patient outcomes from the later period. Although we also observed improvement in a single patient with an interintestinal fistula, the conclusions of isolated cases in a retrospective study should be aired. To provide more robust evidence, larger and prospective studies would be required to assess whether the finding is reproducible and relevant to a wider population. Also, the Institute has only one hyperbaric chamber, which poses scheduling challenges and may have influenced the organization and timing of treatments, while the treatments themselves lasted only 15–20 days. Furthermore, no studies have conclusively demonstrated that a specific duration of HBOT directly affects treatment outcomes. This limitation underscores the need for improved access to hyperbaric therapy infrastructure.

## 5. Conclusions

Hyperbaric oxygen therapy (HBOT), as an adjunctive method in treating CD complications, accelerates recovery, prevents the need for surgery, and shortens hospitalization. Future randomized and prospective studies are needed to further investigate HBOT in this pathology. Still, the experience with HBOT based on the research conducted and the results from the global literature justify the use of HBOT in IBD.

## Figures and Tables

**Table 1:** Demographic and clinical characteristics of patients treated with HBOT.

Category	Total (n = 61)	Conservative (n = 34)	Surgical (n = 27)	*p*-Value
Age, mean (±SD)	32.7 ± 2.3	31.6 ± 2.7	33.2 ± 2.9	0.86
Male sex (n, %)	28 (45.9)	15 (44.1)	13 (44.8)	0.69
BMI, kg/m^2^, mean (±SD)	23.7 ± 4.8	23.9 ± 5.8	23.6 ± 4.2	0.92
Disease type (Greenstein classification)				<0.005
Perforating	18 (29.5%)	6 (17.6%)	12 (44.4%)	
Non-perforating	43 (70.5%)	28 (82.4%)	15 (55.6%)	
Previous surgeries				0.90
Yes	9 (14.7%)	4 (11.7%)	5 (18.5%)	
No	52 (85.2%)	30 (88.3%)	22 (81.5%)	
ASA score				0.92
I–II	55 (90.2%)	32 (94.1%)	23 (85.1%)	
III	6 (9.8%)	2 (5.9%)	4 (14.9%)	
Crohn’s disease complications as a major cause of patient hospitalization				-
Terminal ileitis (moderate to severe)	13	12	1	
Interintestinal fistula	7	5	2	
Mesenteric infiltration	10	8	2	
Small bowel obstruction	12	4	8	
Intestinal bleeding	4	1	3	
Perforation	6	0	6	
Intra-abdominal abscess	9	4	5	

**Table 2:** Primary and secondary outcomes of patients.

Category	Conservative n = 34	Surgical n = 27	*p*-Value
Hospitalization, median (range)	9 (3–25)	12 (6–42)	0.014
Morbidity, n (%)	3 (8.8%)	5 (14.8%)	0.31
Mortality, n (%)	0	0	-
Time to bowel movements, median (range)	2 (1–5)	3 (1–8)	0.56
Need for surgery after HBOT, n (%)	2 (5.8)	2 (7.4)	0.9

**Table 3:** Comparison with patients from the previous five-year period treated without HBOT.

Category	HBOT (n = 61)	Without HBOT (n = 76)	*p*-Value
Age, mean (±SD)	32.7 ± 2.3	31.3 ± 2.6	0.81
Male sex (n, %)	28 (45.9)	37 (48.8)	0.72
Surgical patients	27	29	0.47
Conservative patients	35	47	0.73
BMI, kg/m^2^, mean (±SD)	23.7 ± 4.8	24.2 ± 4.4	0.54
Type of disease (according to Greenstein)			
Perforating	18	28	
Non-perforating	43	48	0.36
ASA score			
I–II	55	64	0.82
III	6	12	0.73
CRP (mg/L), mean (±SD)	67 ± 32.2	94 ± 53	<0.05
Mortality	0	1	-
Need for (re)operation, n (%)	4 (6.5)	14 (18.4)	0.04
Duration of hospitalization, median (range)	11 (3–42)	16 (6–62)	<0.05
Morbidity (>3a)	8 (13.1%)	16 (21.0%)	0.22
Time to the restoration of intestinal passage	2 (1–8)	4 (2–11)	<0.05
Average reduction in CDAI	63 ± 32	51 ± 24	<0.05
Average reduction in van Hees index	112 ± 41	97 ± 39	<0.05

## Data Availability

The data supporting this research are available upon request from the corresponding authors for data protection reasons.
